# Predicting alcohol consumption during the month before and after beginning college

**DOI:** 10.1186/1747-597X-5-11

**Published:** 2010-06-15

**Authors:** Heather R Johnson, William H Zywiak, Daniel D Graney, Robert L Stout, Winston B Trefry, Joy E LaGrutta, Frances C Cohen

**Affiliations:** 1 University of Rhode Island, Office of Student Life, 330 Memorial Union, Kingston, RI 02881, USA; 2 Decision Sciences Institute, Pacific Institute for Research and Evaluation, 1005 Main St., Unit 8120, Pawtucket, RI 02860, USA; 3 Center for Alcohol and Addiction Studies, Brown University, 121 South Main St., Providence, RI 02903, USA

## Abstract

**Background:**

We sought to determine predictors of drinking the month before and after beginning college, as well as changes in drinking between these two periods among adjudicated students. We conducted these analyses to inform individual and university-wide approaches to addressing underage drinking, particularly among the heaviest drinkers.

**Methods:**

The sample consisted of 143 students entering college, adjudicated during their first semester, and interviewed during the same semester. The sample consisted of 43% women. Drinking data were collected through the Time-Line Follow-Back interview.

**Results:**

The average number of drinking days (DD) during the first month of college was 7.0 (SD = 4.7), the average number of drinks per drinking day (DDD) was 7.4 (SD = 3.4), and the average volume of standard drink units consumed during this month was 56.3 (SD = 51.2). Students had volunteered for a two-year college facilitation study, and had been invited to participate after receiving a citation for violating university alcohol policies. Analyses consisted of nine backward elimination regression analyses with nine variables entered as predictors (one was a control variable). Age of first intoxication was related to every dependent measure. Men had a higher August DDD, September DDD, and September volume than women. Roommate drinking level was associated with September DDD and September volume. Out-of-state students had a lower August volume than in-state students. High school rank was inversely related to September drinking days. SAT score, declared major status, and fraternity/sorority status were not related to drinking according to these multivariate analyses.

**Conclusions:**

Results suggest that approaches to underage drinking for adjudicated students may need to be tailored according to age of first intoxication. Results also suggest the drinking level of the heaviest drinking roommate may moderate individual level interventions. Further, interventions applied to an entire dorm room may prove efficacious. Results also suggest that high school rank, rather than SAT scores, should be used as college entry criteria to yield a drier incoming class. Results may not generalize to non-adjudicated students.

## Background

Heavy drinking by college students negatively impact themselves and those around them (including fellow students and local community members). Negative impacts for heavy drinkers include impaired academic performance [1] personal injuries and death (including suicide) [2]. Negative impacts for other persons include sexual assault and physical assault [2]. Perkins [2] adds a third domain of negative impacts: institutional costs. Institutional impacts include property damage, vandalism, student attrition, and the perceived loss of academic rigor [2]. These latter two effects in particular are likely to have long-term financial repercussions for academic institutions, and may even affect the viability of institutions. In spite of these diverse impacts, and despite the efforts of college staff to address heavy drinking, the prevalence of the negative effects of heavy drinking in college increased from 1998 through 2005. These increases were observed in the number of alcohol-related unintentional injury deaths (1,825 per 100,000 students in 2005) and in the percentage driving under the influence in the past year (28.9% in 2005) [3].

Many universities have disciplinary sanctions and/or fines for students observed drinking on campus [4]. This group of students has been found to exhibit greater drinking frequency, quantity, peak drinking, and alcohol-related problems for adjudicated (mandated) students than for the general student population (effect sizes ranged from .20 to .48, where .20, .50, and .80, are small, medium, and large effect sizes, respectively) at this same university [5]. This group is a readily identified group, for whom more efficacious interventions could be developed [6]. The University Assistance Program [7] is one promising option for adjudicated, or mandated students.

A promising line of research for college students in general, consists of the study of specific events and transitions in the context of college drinking [8]. Neighbors et al. reviewing the literature [8] indicate that event-specific strategies targeting sporting events, spring break, and turning 21 have received the most attention. They list the beginning of the school year as another potential time for intervention. Pertinent to this issue, we found that the research studies on drinking pre and post college entry primarily examined averages across years or large intervals, see for example [9]. While Del Boca and colleagues gathered fine-grained drinking data (number of drinks per calendar day) and conducted fine-grained statistical analyses, they focused on the first semester, but did not examine the period before the semester in their analyses [10]. Sher and Rutledge [11] did examine drinking before and after the onset of college but they collected data regarding the number of days high/light headed, drunk, and/or drinking 5 or more drinks in the last 30 days, but did not collect the level of detail that Del Boca and colleagues did. We took the strengths of these two approaches and combined them.

We have collected drinking data using the Time-Line Follow-Back [12] inquiring about the last 6 months. In this semi-structured interview, information is collected so that standard drink units can then be calculated and tagged to every single day in the time period queried. In the present paper, we zero in on drinking behavior during the month before and after one salient transition for young adults, entering college. We have collected detailed drinking calendar data on substance use policy offenders of a large state university in the Northeast. We have used this fine-grained data to examine predictors of alcohol consumption during the first month of college, the month before the first month of college, and increases in drinking across these two periods.

## Methods

First Offenders of the University's Alcohol and Drug Policy were invited to participate in the College Facilitation 3 Study. This study is a large randomized trial of College Facilitation. College Facilitation consists of 18 planned telephone contacts once a month during the academic year for a total of two calendar years. The tone of the calls is non-confrontational, supportive, and empathic. College Facilitation is designed to reduce drinks per drinking day, to reduce peak drinking levels, and to reduce the frequency of drinking related consequences within the context of helping the student get the most out of college, both academically and as a well-rounded person. The College Facilitation protocol is based on the Extended Case Monitoring protocol [13,14] which, in turn, draws heavily from the Rogerian [15] client-centered approach. Promising College Facilitation pilot study results for drinks per drinking day and alcohol-related negative consequences have been presented [16]. This larger randomized trial aims to capitalize on the nearly unique opportunity afforded by college students being available for a number of years, just when drinking may be escalating. To be eligible for this study, First Offenders had to be at least 18 years old, had to have at least two heavy drinking days in the last 29 days (4 drinks for women, 5 for men), and had to have at least one alcohol-related negative consequence in the last 12 months (e.g., missing class, saying something s/he regretted, or throwing up).

This research was carried out in compliance with the Helsinki Declaration and is approved by the Institutional Review Boards of the Pacific Institute for Research and Evaluation and the university where the study was conducted. After providing documented informed consent, students completed an interview that included closed-ended questions, open-ended questions, surveys, and the semi-structured Time-Line Follow-Back. We closed participant recruitment in September 2007 with a sample of 374. This analysis focuses on Freshmen recruited during the Fall 2005 and 2006 semesters. During the course of recruitment approximately 20 adjudicated students had less than two heavy drinking days in the last 28 days, and were therefore ineligible. Approximately 16 students that were eligible were not interested in participating in the study. One student was underage when recruited, and s/he was not interested in the study after s/he turned 18.

All but 16 of the 143 freshmen in the sample had at least 28 days of data following the opening of the dorms. The duration of the data for the other 16 ranged from 23 to 27 days, and their drinking volume and number of drinking days were prorated to make up for the shorter assessment window. Participants for the results presented here consist of college students beginning their first semester of college and interviewed between September 26, 2005 and December 19, 2005 or between September 25, 2006 and December 7, 2006 (inclusive). Dormitories opened on Saturday September 3, 2005 and September 2, 2006. We operationalized the first month of college as the first 28 days starting with the opening of the dormitories. (Advising Day was scheduled on Tuesday September 6, 2005 and September 5, 2006. Classes began on Wednesday September 7, 2005 and September 6, 2006.) We used an integer number of weeks to control for the variation in drinking during the week [10]. We operationalized the month before the beginning of college as the 28 days ending on the Friday before the dormitories opened. Only baseline data were used in these analyses. These data were collected just prior to participants being randomly assigned, through urn randomization, to College Facilitation or college as usual. We excluded data from students completing baseline interviews in the Spring semester to reduce retrospective bias. Drinks per drinking day (DDD), total volume (in standard drink units), and number of drinking days (DD) were chosen as the variables of interest since they parsimoniously and comprehensively describe the drinking behavior of the students. For example, a student who has 1 drink a day for 24 days and another student who has 24 drinks on a single day, will have identical volumes, but different DDs and DDDs. A student that has six drinks on a single day, and another student that has six drinks every day in a month will have identical DDDs, but different DDs and volumes. Drinking days, volume, and DDD were crossed by month (August and September) and a change score, resulting in nine dependent variables. The change score reflects an increase, e.g., September drinking days less August drinking days. During August, 4% of the sample was abstinent, and 2% drank every day. During September, 1% of the sample was abstinent and 1% drank every day.

The eight hypothesized predictor variables can be grouped into pre-collegiate and collegiate variables. Pre-collegiate variables, in turn, can be grouped into demographics (sex, Rhode Island residency status), academic variables (high school rank, SAT score), and drinking history (age of first intoxication). Collegiate variables include fraternity status, whether the student has declared a major, and the heaviest drinking status of the student's roommate(s). These variables can also be used to describe the sample of 143. The sample consisted of 43% women. (The incoming class of 2006 was 56% women.) The average age of first intoxication was 15.7 (SD = 1.4). Age of first intoxication ranged from 12 to 18 years old. The average high school rank was the 70th percentile (SD = 15%; where a higher percentile reflects higher grades). The mean SAT score was 1130 (SD = 120) out of a possible 1600 points, and 29% of the students were in-state residents. Sixty nine percent of the students had declared a major. Eight percent were fraternity/sorority members or pledges. All participants had at least one roommate. Participants were asked whether their roommate was a heavy, moderate, or light drinker, or abstainer. If a student had more than one roommate, the heaviest drinking roommate was counted. Five percent reported that their heaviest drinking roommate was an abstainer, 18% reported a light drinker, 64% reported a moderate drinker, and 13% reported that their heaviest drinking roommate was a heavy drinker. We also included one control variable: 39% of the participants were written up during the September window. (None of the participants were written up during the August window.) Most (61%) were written up after the windows of drinking data presented here. All of the data presented were based on self-report. Participants were compensated $25 for their participation in the baseline assessment.

## Results

An examination of the distributional characteristics of the drinking variables revealed that these variables approximated the normal distribution, in contrast to DDD and DD observed in clinical samples [17]. Paired t-tests revealed statistically significant reductions in DDD and volume from August to September. Correlations for each drinking variable from time 1 to time 2 ranged from .54 to .57. T-tests for homogeneity of variance [18] revealed a constriction in variance (less variation) for drinking days and volume from time 1 to time 2. (Please see Table 1.) September volume was correlated with September drinking days [r(142) = .84, p < .001] and September drinks per drinking day [r(142) = .64, p < .001]. September drinking days were in turn, correlated with September drinks per drinking day [r(142) = .25, p = .003].

**Table 1:** Statistics on drinking variables (n = 143)

	**August**	**September**		**means:**		**variance**	
	**M (SD)**	**M (SD)**	**r (p)**	**paired t(142)**	**p**	**t(141)**	**p**
			
DD	7.8 (6.5)	7.0 (4.7)	.54 (.001)	1.54	.13	3.29	< .01
Volume	76.4 (100.6)	56.3 (51.2)	.55 (.001)	2.85	< .01	7.85	< .001
DDD	8.1 (4.1)	7.4 (3.4)	.57 (.001)	2.32	< .05	1.59	< .10

The eight predictor variables and one control variable were entered into a backward stepwise regression analysis for each of the nine dependent variables. Age of first intoxication was related to every dependent measure. Men had a higher August DDD, September DDD, and September volume than women. Roommate drinking level was associated with September DDD and September volume. Out of state students had a lower August volume than in-state students. Out of state students showed a marginally significant increase in volume, relative to in-state students. High school rank was inversely related to September drinking days (better grades less drinking). High school rank was marginally inversely related to September volume. High school rank was marginally related to a decrease in drinking days. On the other hand, SAT score, declared major status, and fraternity/sorority status were not related to drinking according to these multivariate analyses (i.e., when other variables were accounted for). If the students were cited for violating university policy in September (as opposed to later in the first semester) the students were marginally likely to have a greater August volume, and were drinking on more days in September. [Please see Table 2 for regression statistics and individual predictor statistics.] (Age of first drink was a tenth predictor in earlier models, but was dropped due to collinearity with age of first intoxication, and since age of first intoxication was more strongly related to the first six dependent measures than age of first drink.)

**Table 2:** Regression statistics, standardized betas and t-tests by predictor and dependent variables

	August			September			Increase		
	DD	Vol.	DDD	DD	Vol.	DDD	DD	Vol.	DDD
	
F-value	50.58	17.68	31.81	13.28	16.49	20.40	6.43	9.00	8.28
df_n_/df_d_	1/141	3/139	2/140	3/139	4/138	3/139	2/140	2/140	1/141
p-value	.001	.001	.001	.001	.001	.001	.002	.001	.005
Adj. R^2^	.26	.26	.30	.21	.30	.29	.07	.10	.05
Standardized Betas:									
									
residency		-.17*						.16†	
HS Rank				-.18*	-.14†		-.15†		
Roommates					.21**	.19**			
sex			-.38***		-.23**	-.44***			
offense		.14†		.15*					
1stIntox	-.51***	-.53***	-.45***	-.37***	-.42***	-.28***	.28**	.35***	.24**

t-values									
residency		2.29						1.87	
HS Rank				2.28	1.92		1.82		
Roommates					2.91	2.70			
sex			5.37		3.20	6.20			
offense		1.88		2.00					
1stIntox	7.11	7.04	6.41	4.86	5.85	3.97	3.42	4.18	2.89

*** denotes p < .001; ** denotes p < .01, * denotes p < .05, † denotes p < .10

**Figure 1:**
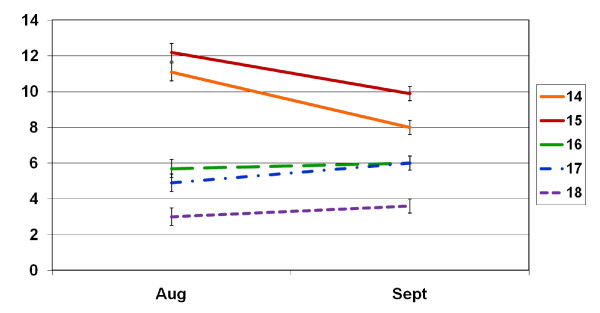
Drinking Days by Age of First Intoxication

August and September drinking days by age of first intoxication are plotted in Figure 1. Descriptively, those with a heavy drinking roommate had a September DDD of 9.6 (SD = 6.0), a moderate drinking roommate a DDD of 7.3 (2.6), a light drinking roommate a DDD of 6.5 (2.8) and an abstinent roommate a DDD of 6.3 (2.6). T-tests paralleling the regression findings replicated the significant and pronounced sex effects. On average, men drank 9.29 drinks per drinking day in August (SD = 4.01) compared to 6.55 DDD (SD = 3.66) for women [t(142) = 4.24, p < .001]. Men drank 8.67 drinks per drinking day in September (SD = 3.48) compared to 5.78 DDD (SD = 2.38) for women [t(142) = 5.87, p < .001]. For September volume, men drank 65.1 drinks (SD = 52.7) compared to women's 44.4 drinks [SD = 46.9, t(142) = 2.44, p = .02].

A t-test for August volume and residency was not significant, but a t-test for age of intoxication and residency revealed that out of state students [15.50 (1.35)] were first intoxicated almost a year younger on average than in-state students [16.33 (1.18), t(141) = 3.50, p = .001] indicating that residency does predict August volume when age of first intoxication is taking into account through the multivariate analysis.

## Discussion

The reductions in drinking volume and DDD from August to September were initially surprising, given the literature's focus on problematic alcohol use during college. Future research should investigate whether this finding is specific to adjudicated students or generalizes to Freshmen in general. While some studies [19,20] have shown alcohol reductions subsequent to being written up for violating a school's policy, this effect does not seem to account for the reduction in drinking since this variable did not predict any of the three drinking change variables.

The reductions in drinking volume and DDD from August to September suggest that parents [21,22] share the responsibility of the heavy drinking college student with Universities and the students themselves. This finding also suggests that campus-wide interventions may best be targeted very early on (e.g., September of Freshman year). Variance was greater for August DD and volume than for the September DD and volume, suggesting that methodologically, it may be important to distinguish between drinking between the first day of class and before the last exam or paper submitted, and drinking outside of this "semester" when examining intervention effects. It should be noted that while the beginning of the semester is clear-cut across the student sample, the end of the semester varies and will require additional data collection to be operationalized. This difference in variation between August and September drinking may be a function of greater difficulty in recalling, and therefore greater error variance associated with more distal drinking.

While high school rank and SAT scores are correlated with each other, only high school rank is related to September drinking in this sample. SAT scores reflect academic aptitude and high school rank reflects actual academic performance. The regression results suggest that high school rank would be a useful university eligibility criterion that could also lead to a drier student population, whereas SAT scores are not a useful criterion in this regard.

Students with an earlier first intoxication had greater drinking levels in August and September than students with a later first intoxication. However, students with an earlier first intoxication showed decreases in drinking from August to September, while students with a later intoxication showed increases in drinking during this time period. There is a convergence in drinking behavior across the sample as indicated by the constriction in variance (a reduction in variability) across drinking days and volume. Access to alcohol may vary widely across the participants in August (e.g., residences with a bar in the basement versus dry households) but will be more uniform in September since most if not all the Freshmen lived in the dorms. For students with an earlier age of first intoxication the supervision of a residence hall advisor may reflect a healthier environment than the one they left. For students with a later age of intoxication, the college environment may reflect a less structured environment than the one they left. Figure 1 further suggests that two different types of intervention strategies may be needed for students who have already acquired heavy drinking behavior and those that are more recently increasing their drinking. For example, increasing the salience of already experienced alcohol-related negative consequences, and assessing family history may be important for those with an earlier age of intoxication. On the other hand, reinforcing positive academic achievement, and possibly changing roommates, may be important for those with a later age of first intoxication. These findings also suggest that college facilitation may have a bigger effect on those with a later onset of drinking and intoxication, because they are more recently acquiring the behavior. The drinking status of the heaviest drinking roommate predicted September drinking but not August drinking. Results suggest that interventions may be usefully demarcated by room rather than individuals. As far as implementing a randomized trial, this may indicate that if roommates happen to be in a study together, it may be appropriate to assign the second student to the condition the first student was randomly assigned to. In another vein, a peer-based intervention [23] could be provided by the roommate that is the lighter drinker to the roommate that is the heavier drinker, especially if the lighter drinking roommate is enrolled in a psychology, social work, or similar program.

There are a number of caveats and limitations with the present findings. These results may not generalize to all universities. Reflecting the demographics of this university (with 27% minority students) coupled with the risk for heavy drinking among minority students, minorities were not well represented in this sample (4%). All of the data (including drinking data) are self-report. Most of the predictor variables utilized in these analyses may be conceptualized as demographic variables. Variables such as alcohol expectancies and decisional balance, known to be related to drinking variables, were not examined. Further, the analyses do not examine potential moderators (e.g., sex). The null finding regarding fraternity/sorority status should be treated with caution. Only 8% of the sample consisted of members or pledges, so we had insufficient statistical power to detect a difference on this variable. Further, since we recruited students early on, they may have not had the opportunity to pledge or join fraternities and sororities. While representative of both sexes, the sample is not representative of minorities. While the roommate's drinking was a predictor, we are unable to tease out selection versus socialization. We did not ask if the participant knew, or drank with, their roommate before beginning college.

## Conclusions

This study has policy, prevention, and intervention implications. Results suggest that approaches to underage drinking for adjudicated students may need to be tailored according to age of first intoxication. Results also suggest the drinking level of the heaviest drinking roommate may moderate individual level interventions. Further, interventions applied to an entire dorm room may prove efficacious. Results also suggest that high school rank, rather than SAT scores, should be used as college entry criteria to yield a drier incoming class. Age of first intoxication predicted all nine dependent measures, and is an important possible point for prevention efforts, and an important variable in college drinking research. Sex is an important variable in college drinking research, especially in regard to drinks per drinking day.

## List of Abbreviations

1stIntox: age of first intoxication, DD: drinking days, DDD: drinks per drinking day, HS: high school, M: mean, SAT: Scholastic Aptitude Test, SD: standard deviation, Vol: volume.

## Competing interests

The authors declare that they have no competing interests.

## Authors' contributions

HRJ recruited, interviewed and administered surveys to participants, entered data, and provided substantial input on the manuscript. WHZ is the PI of the NIAAA grant, has overall primary oversight of the day to day operations of this project, conducted the statistical analyses, wrote the first draft of the manuscript, and was responsible for the editing of the final draft. DDG has primary oversight of HRJ and provided substantial input on the manuscript. RLS assisted with conceptualizing the design of the overall study, assisted with finalizing the statistical plan of the overall study, and provided substantial input on the manuscript. WBT and JEL provide the college facilitation telephone contacts and provided substantial input on the manuscript. FCC was the liaison to the Vice President of Student Affairs, and provided substantial input on the manuscript. All authors read and approved the final manuscript.
